# High-risk extended-spectrum β-lactamase-producing Klebsiella pneumoniae ST307 in a neonatal sepsis outbreak in Zambia: a secondary genomic analysis

**DOI:** 10.1099/acmi.0.001164.v4

**Published:** 2026-10-12

**Keywords:** extended-spectrum β-lactamase, *Klebsiella pneumoniae*, mobile genetic elements, neonatal sepsis, plasmid-mediated resistance, ST307

## Abstract

Neonatal sepsis caused by multidrug-resistant *Klebsiella pneumoniae* contributes substantially to neonatal morbidity and mortality, especially where treatment options and genomic surveillance are limited. Phenotypic microbiology defines susceptibility, whereas Illumina short-read whole-genome sequencing (WGS) links phenotypes to lineage, capsule, resistome, virulome and mobilome contexts. However, complete characterization of mobile genetic element (MGE) structure remains inferential. This secondary analysis re-examined 411 public *K. pneumoniae* genomes from the University Teaching Hospital neonatal outbreak in Zambia. The population was dominated by ST307/KL102/OL2α.2, a comparatively recent but globally expanding high-risk clone, accounting for 284 out of 411 genomes (69.1%). Minority genomes included established resistance or virulence-associated high-risk backgrounds, including carbapenemase-positive KL15/OL4 lineages and yersiniabactin-positive ST147/ST985. Extended-spectrum β-lactamase (ESBL) genotypes were widespread (396 out of 411; 96.4%) and mainly driven by *bla*CTX-M-15 (406 out of 411; 98.8%), whereas carbapenemase carriage was restricted to *bla*NDM-5-positive ST5856/ST340 (11 out of 411; 2.7%). Plasmids were the principal acquired resistance layer, as most AMR genes were plasmid-associated, and similarity analysis revealed a cohesive ST307-linked IncFIB/IncFII AA277 ESBL background, in contrast to distinct minority ESBL and IncX3 *bla*NDM-5 architectures. This pattern supports clonal maintenance of a stable ESBL plasmid background within ST307, while identifying IncX3 *bla*NDM-5 plasmids in minority lineages as a key convergence risk. Resistance genes were further structured by integron cassettes, insertion-sequence modules and plasmid cargo containing metal-, biocide- or stress-response genes. Virulence was limited and mainly ICEKp/ybt-associated, and widespread predicted prophages did not carry priority determinants. These findings show that WGS can add mechanistic resolution to routine phenotypic surveillance by identifying clone–MGE configurations that warrant targeted monitoring in neonatal *K. pneumoniae* outbreaks in Zambia.

**Impact Statement**

Neonatal sepsis caused by multidrug-resistant *Klebsiella pneumoniae* is difficult to treat and can be especially dangerous in hospitals where laboratory and genomic surveillance capacity are limited. Routine antibiotic susceptibility testing indicates which drugs an isolate is likely to resist, but it does not show how resistance spreads among bacterial strains or whether resistance genes are carried on mobile DNA that may move between lineages. This study re-analysed publicly available genomes from a neonatal sepsis outbreak in Zambia to show how whole-genome sequencing can add this missing layer of information. The analysis found that the outbreak was dominated by ST307, a globally expanding high-risk *K. pneumoniae* clone, but also showed that resistance was strongly linked to plasmids and other mobile genetic elements rather than to the clone alone. This distinction matters because a dominant hospital-adapted clone may become more difficult to control if it acquires plasmids carrying carbapenemase genes, which can compromise last-line antibiotic options. These findings support the use of genomic surveillance that tracks not only bacterial lineages, but also the mobile resistance elements they carry. For neonatal units in Zambia and similar settings, this approach could help detect emerging high-risk clone–plasmid combinations earlier, guide infection-prevention priorities and strengthen antimicrobial stewardship decisions.

## Data Summary

The author confirms that all supporting data, code and protocols are provided within the article or in supplementary data files. Raw Illumina read data re-analysed in this study were obtained from draft genome submissions originally deposited by Phillips *et al*. [1] and are publicly available via the European Nucleotide Archive (ENA) under BioProject PRJEB46513 (https://www.ebi.ac.uk/ena/browser/view/PRJEB46513). Run accessions and sample identifiers are listed in Table S1. The reproducible analysis workflow (scripts, parameters and software versions) and key intermediate outputs required to regenerate summary tables and figures are provided in https://figshare.com/articles/dataset/Klebogenomics_Code_Scripts/30927326, together with README and VERSIONS.txt files describing the computational environment (https://figshare.com/articles/dataset/Metadata_Files/30927395).

## Introduction

*Klebsiella pneumoniae* is a Gram-negative bacterium in the order Enterobacterales. It acts as both a gut commensal and a significant opportunistic pathogen, being responsible for pneumonia, urinary tract infections and, most notably in this context, bloodstream infections and sepsis [2]. Two pathotypes are recognized: hospital-associated classical *K. pneumoniae*, typically multidrug-resistant (MDR) and of moderate virulence, and hypervirulent *K. pneumoniae* (hvKp), which carries accessory virulence loci and causes severe community-acquired disease, although convergent MDR hypervirulent strains are increasingly reported [3]. The polysaccharide capsule is the foremost virulence determinant, conferring resistance to complement-mediated serum killing and phagocytosis, thereby facilitating bloodstream survival, invasion and dissemination [3, 4]. In the 2024 Bacterial Priority Pathogens List, the World Health Organization (WHO) identified carbapenem-resistant *K. pneumoniae* as one of the leading priority pathogens. It classified carbapenem-resistant and third-generation cephalosporin-resistant Enterobacterales as a critical priority, reflecting their substantial burden in low- and middle-income countries (LMICs) [5].

Neonatal sepsis remains a major cause of death in the first month of life, with the greatest burden falling on LMICs. In the BARNARDS study, clinically suspected sepsis was estimated at 166 episodes per 1,000 live births in these settings, where antimicrobial-resistant Gram-negative bacteria increasingly dominate neonatal sepsis pathogens [6]. The WHO first-line regimen of ampicillin plus gentamicin, with cephalosporin escalation, is steadily eroded by extended-spectrum β-lactamase (ESBL) production and aminoglycoside-modifying enzymes, and the WHO has specifically identified ESBL-producing *K. pneumoniae* as complicating neonatal sepsis treatment in LMICs [2, 5, 7]. Neonates are highly susceptible to invasive infection because innate and adaptive immunity are still developing, and complement activity is reduced. In neonatal intensive care units, this vulnerability is compounded by gastrointestinal colonization, invasive devices, shared care and antimicrobial exposure, favouring nosocomial transmission and clonal amplification [1, 8, 9]. Outbreaks of *K. pneumoniae* in African neonatal units, including in Zambia, are well documented [1, 10–13]. Resistance in *K. pneumoniae* combines intrinsic and acquired mechanisms. The species is intrinsically resistant to aminopenicillins due to a chromosomal class A SHV β-lactamase, so ampicillin resistance is assumed based on species identification and is not routinely tested or reported [14, 15]. Acquired resistance most often follows, most often through an ESBL, overwhelmingly the CTX-M-15 enzyme encoded by *blaCTX-M-15*, which inactivates penicillins and oxyimino-cephalosporins [16–19]. Carbapenems then become the practical last line and are, in turn, threatened by carbapenemases, including the New Delhi metallo-β-lactamase (NDM), of which *blaNDM-5* is an increasingly common and highly active variant. As a metallo-β-lactamase, NDM is not inhibited by the serine-β-lactamase inhibitors tazobactam or avibactam, so that β-lactam and β-lactamase-inhibitor combinations remain ineffective against NDM producers [20]. Resistance accumulates across classes, producing MDR and extensively drug-resistant phenotypes [21]. Further resistance arises by chromosomal mutation, including quinolone resistance-determining region (QRDR) changes in *gyrA* and *parC* and loss of the outer membrane porins OmpK35 and OmpK36, which can raise carbapenem minimum inhibitory concentrations even without a carbapenemase [22–24].

Most acquired antimicrobial resistance (AMR) determinants in *K. pneumoniae* are carried or mediated by mobile genetic elements (MGEs), particularly plasmids, integrons, insertion sequences (ISs) and transposon-associated regions [25, 26]. Conjugative and mobilizable plasmids, classified by incompatibility (Inc) replicon types such as the IncF and IncX families, are the principal vehicles in which resistance genes are nested within smaller elements [26, 27]. Class 1 integrons capture and rearrange gene cassettes, while ISs and the composite transposon mobilise individual genes and supply promoters [25, 28]. Prophages are abundant and diverse in *K. pneumoniae* [29]. However, acquired resistance in this species is mainly associated with other MGEs. Prophages contribute more to genome diversity and horizontal gene transfer than to AMR [30–32]. A further consideration is co-selection, whereby antibiotic-resistance genes carried on the same plasmid as heavy-metal or biocide-resistance genes are co-maintained under metal or disinfectant exposure even without antibiotic pressure [33, 34].

The epidemiology of resistant *K. pneumoniae* is dominated by globally disseminated high-risk clones, defined by MLST as sequence types (STs). ST307 is among the most important, having spread internationally as an ESBL clone associated with *blaCTX-M-15* and increasingly acquiring carbapenemases [29, 35, 36], while ST147, ST101 and clonal complex 258 further contribute to the carbapenemase burden [37]. Lineages are also defined by surface antigens: the capsule at the K locus and the LPS O antigen at the O-locus, which are typed *in silico* as KL and OL types [38]. A small set of capsule types, notably K1 and K2, is associated with hypervirulence and invasive disease. However, the hypervirulence phenotype depends on accessory aerobactin, salmochelin, colibactin and *rmp* loci rather than capsule type alone, and yersiniabactin, usually carried on an integrative and conjugative element (ICEKp), is a weaker marker.

Short-read Illumina sequencing dominates *K. pneumoniae* outbreak and surveillance genomics due to its accuracy, scalability and cost [39, 40]. However, it cannot fully resolve repetitive or complex regions such as ISs, transposon boundaries, complete plasmids or capsule-locus rearrangements. Plasmid reconstruction and gene co-localization from short-read assemblies should therefore be read as strong inferences about genomic context rather than definitive evidence of structure or transfer [41].

This study is a secondary genomic analysis of a neonatal *K. pneumoniae* outbreak at the University Teaching Hospital (UTH) in Lusaka, Zambia. The primary investigation, the Sepsis Prevention in Neonates in Zambia (SPINZ) study, characterized the outbreak in terms of strain epidemiology and transmission, documenting ST307 predominance, the resistome and virulome, capsule and O-locus types and transmission structure, along with initial plasmid data [1]. Building on those findings, the present analysis addresses the finer-scale MGE architecture that organizes resistance, which the transmission-focused primary study only partially addressed. While that study grouped plasmids into clusters, it did not examine how resistance genes are nested within integrons and ISs on those plasmids, whether they co-occur with metal-, biocide- or stress-resistance genes or whether they overlap with prophages. The present analysis thus re-examined all 411 genomes using the same short-read assemblies to address the MGE architecture specifically. To the best of current knowledge, this represents the first integrated clone-, capsule-, plasmid- and mobile-element-resolved reanalysis of Zambian neonatal *K. pneumoniae* and is intended to complement, rather than duplicate, the transmission-focused findings of the primary study.

## Methods

### Study design and data source

This retrospective secondary genomic analysis utilized publicly available whole-genome sequencing data from the SPINZ study [1], which investigated *K. pneumoniae* transmission and infection prevention in the UTH neonatal unit in Lusaka, Zambia. The current analysis focused on characterizing lineage structure, capsule-locus architecture, the resistome and virulome, plasmid-associated resistance context and MGE organization, without reassessing infection-prevention interventions. Raw paired-end Illumina reads were obtained from the European Nucleotide Archive under BioProject PRJEB46513. A total of 411 paired-end Illumina read sets were retrieved, each linked to a unique sequencing-run identifier and corresponding sample metadata. Each read set was assembled independently, and the resulting genome assembly was used as the unit of analysis.

### Genome assembly and quality control

Read quality was assessed using FastQC v0.12.1 and aggregated with MultiQC v1.34 [42, 43]. Read sets were inspected for per-base and per-sequence quality, adapter and overrepresented-sequence content and ambiguous bases. Module flags were interpreted in context rather than applied as automatic exclusion criteria, and a read set was considered eligible for assembly when both paired files were present, readable and non-empty, and no critical read-level failure was identified. *De novo* assembly was performed using Unicycler v0.5.1, which uses SPAdes for short-read bacterial genome assembly and constructs a graph for each run [44, 45]. Post-assembly quality was assessed with QUAST v5.3.0 [46], and genome retention followed the KlebNET-GSP framework used by the primary study, requiring fewer than 500 contigs, a total assembly length of 4,969,898–6,132,846 bp and a G+C content of 56.35–57.98 mol%, with downstream confirmation as *K. pneumoniae* or the *K. pneumoniae* species complex [1, 47]. All 411 read sets yielded non-empty assemblies that passed quality control and were retained.

### Species confirmation and typing

Species confirmation, MLST and lineage assignment were performed using Kleborate v3.2.4 and MLST v2.33.1 [40, 48]. Capsule (K) and O-antigen loci were typed using Kaptive v3.2.1.36. ST, K-locus, predicted K type, O-locus, confidence metrics and problem flags were integrated into combined ST/K-locus/O-locus lineage groups. Because ST307 genomes with disrupted canonical K-locus 102 (KL102) calls may carry an alternative capsule-associated locus, all assemblies were screened for Cp2/Cps2 by blastn v2.16.0+ against the 14,020 bp reference sequence KY271410.1 [1, 49, 50]. A genome was classified as Cp2/Cps2-positive when the reference was recovered as a full-length or near-complete match with high identity. Cp2/Cps2 positivity was interpreted as evidence of an alternative capsule-associated locus, not as a canonical K-locus assignment or proof of capsule expression.

### AAMR and virulence genotyping

Acquired AMR determinants were characterized using Kleborate v3.2.4, which provides *Klebsiella*-specific resistance screening and genotype-based resistance scoring, and AMRFinderPlus v4.2.7, using database version 2026-03-24.1, which provides coordinate-resolved gene calls retained for downstream plasmid and MGE localization analyses [40, 51]. ABRicate v1.4.0 was used with the NCBI, ResFinder and CARD databases to cross-check priority resistance determinants [51–53]. Kleborate resistance and virulence scores were used as ordinal summaries of genotypes. Resistance scores range from 0 to 3: **0**, no ESBL/carbapenemase; **1**, ESBL without carbapenemase; **2**, carbapenemase without colistin resistance; and **3**, carbapenemase plus colistin resistance. Virulence scores range from 0 to 5 based on acquired virulence loci: **0**, none of yersiniabactin/colibactin/aerobactin; **1**, yersiniabactin only; **2**, colibactin without aerobactin; **3**, aerobactin only; **4**, aerobactin plus yersiniabactin without colibactin; and **5**, yersiniabactin, colibactin and aerobactin together. Chromosomal resistance-associated variants were retained separately, with attention to QRDR mutations in *gyrA* and *parC*, plasmid-mediated quinolone resistance genes, ciprofloxacin genotype predictions and OmpK35 and OmpK36 changes, the latter treated as permeability-associated modifiers rather than acquired genes. Virulence loci, including yersiniabactin, colibactin, aerobactin, salmochelin and *rmp*, were assessed with Kleborate, and the virulence score was interpreted accordingly. Virulence outputs were integrated with lineage and AMR data to identify convergence of resistance and virulence, particularly in ESBL- or carbapenemase-positive lineages carrying yersiniabactin.

### Plasmid context, mobility and same-contig cargo

Plasmid reconstruction and typing were performed using MOB-suite v3.1.9, which classified contigs as plasmid-associated or chromosomal and assigned replicon type, relaxase type, mating-pair-formation type, predicted mobility and primary and secondary cluster identifiers [54]. Antimicrobial-resistance gene localization was inferred by linking AMRFinderPlus coordinate-resolved gene calls to MOB-suite contig classifications, yielding plasmid-associated, chromosomal or unresolved categories, which were treated as contig-level computational predictions rather than complete plasmid reconstructions [51, 54, 55]. Localization was summarized by lineage, and dominant AMR-associated clusters were identified by counting plasmid-associated hits per MOB-suite primary cluster. Heavy-metal, biocide and stress-associated loci were summarized separately from direct antibiotic-resistance determinants to avoid conflating broader adaptive cargo with AMR. Strict same-contig co-localization counted AMR, metal, biocide, stress and virulence-associated genes only when present on the same plasmid-associated contig within the same genome and was interpreted as genomic co-occurrence rather than as evidence of functional co-selection. Recurrent priority plasmid clusters (AA277, AA276, AA038, AA275, AA405, AA019, AA454, AA816 and AC899) were compared using Mash v2.3, with pairwise distances and connected components recorded at thresholds of 0.005, 0.01 and 0.02 [56]. Mash similarity was interpreted as sequence-relatedness among reconstructed plasmid sequences rather than as evidence of direct plasmid transmission.

### Targeted MGE context

Targeted MGE context analysis was performed for priority antimicrobial-resistance, yersiniabactin-associated and capsule-associated loci. Integrons and gene cassettes were identified using IntegronFinder v2.0.6, and AMRFinderPlus coordinate-resolved gene calls were intersected with IntegronFinder intervals using the same-contig coordinate overlap logic [25, 51]. A gene was counted as integron-associated only when its coordinates directly overlapped a predicted integron or cassette interval on the same contig. ISs were predicted using ISEScan v1.7.3, and priority genes were classified as IS-overlapping, IS-proximal when the nearest IS occurred within 5,000 bp or same-contig-only when an IS was present on the same contig but outside the proximity threshold [28]. Candidate composite-transposon-like contexts were assessed using 20 kb flanking windows around priority genes, with calls ranging from no IS, through insertion-sequence presence without clear flanking architecture, to flanking ISs of different types or within the same family or cluster [28, 57]. These calls were interpreted as computational indicators of possible local mobilization architecture rather than confirmation of complete functional transposons. Prophage/provirus regions were predicted using geNomad v1.12.0 with database v1.9 and default post-classification filters, including the minimum virus/plasmid score of 0.7 and hallmark-gene support for sequences <2,500 bp. Priority genes were considered provirus-associated only when their coordinates directly overlapped predicted provirus intervals on the same contig [58]. Yersiniabactin markers such as *ybtP* and *ybtQ* were classified as ICE/ybt-associated from Kleborate outputs and were not treated as plasmid-associated antimicrobial-resistance determinants.

## Results

### Genome dataset

All 411 paired-end read sets satisfied read-level quality criteria, and all reassembled genomes passed KlebNET-GSP assembly quality control. Consequently, no samples were excluded, and the complete set of 411 genomes was retained for analysis. The resulting outputs were integrated into a sample-level dataset encompassing genome quality, lineage, capsule-locus status, AMR and virulence genotypes, as well as plasmid and MGE context (Fig. 1).

**Fig. 1.**
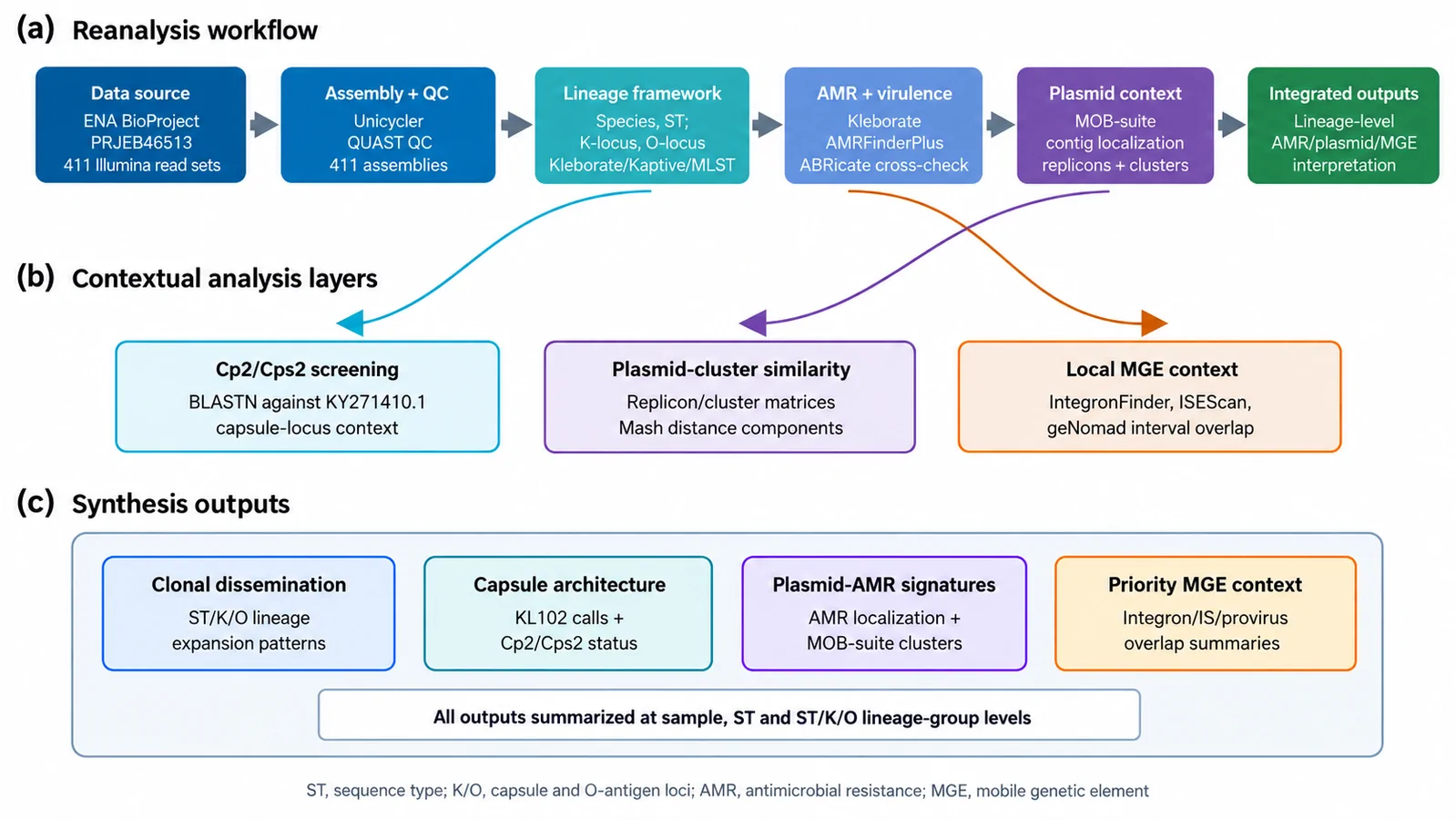
An integrated genomic workflow used for secondary analysis of the *K. pneumoniae* outbreak dataset. (**a**) Reanalysis workflow from publicly available read data through assembly, typing, AMR/virulence genotyping, plasmid-context inference and integrated lineage-level outputs. (**b**) Contextual analytical layers used to refine capsule-locus, plasmid-cluster and local MGE interpretation. (**c**) Main synthesis outputs used to compare clonal dissemination, capsule architecture, plasmid-associated AMR signatures and priority MGE context across sample, ST and ST/K/O lineage-group levels. K/O, capsule and O-antigen loci.

### Population structure

The population was dominated by ST307/KL102/OL2α.2, accounting for 284 out of 411 genomes (69.1%) (Fig. 2). The next most frequent profiles were ST2004/KL23/OL2α.2 (58; 14.1%) and ST983/KL127/OL13 (12; 2.9%), with ST5856/KL15/OL4 and ST101/KL17/OL2α.1 each at nine (2.2%), ST985/KL39/OL2α.2 at six (1.5%) and ST147/KL64/OL2α.1, ST15/KL54/OL2α.2 and ST1119/KL110/OL3γ each at four (1.0%), with the remaining profiles totalling 21 (5.1%). More than two-thirds of the dataset therefore belonged to a single ST307 background, while the remaining 127 genomes (30.9%) represented minority profiles carrying distinct AMR, virulence, plasmid or MGE signatures. The strict ST307/KL102/OL2α.2 definition deliberately excluded a single-locus variant of ST307 (ST307-1LV), which differs from ST307 at one of the seven MLST loci and was therefore counted among the minority profiles rather than within the dominant lineage.

**Fig. 2.**
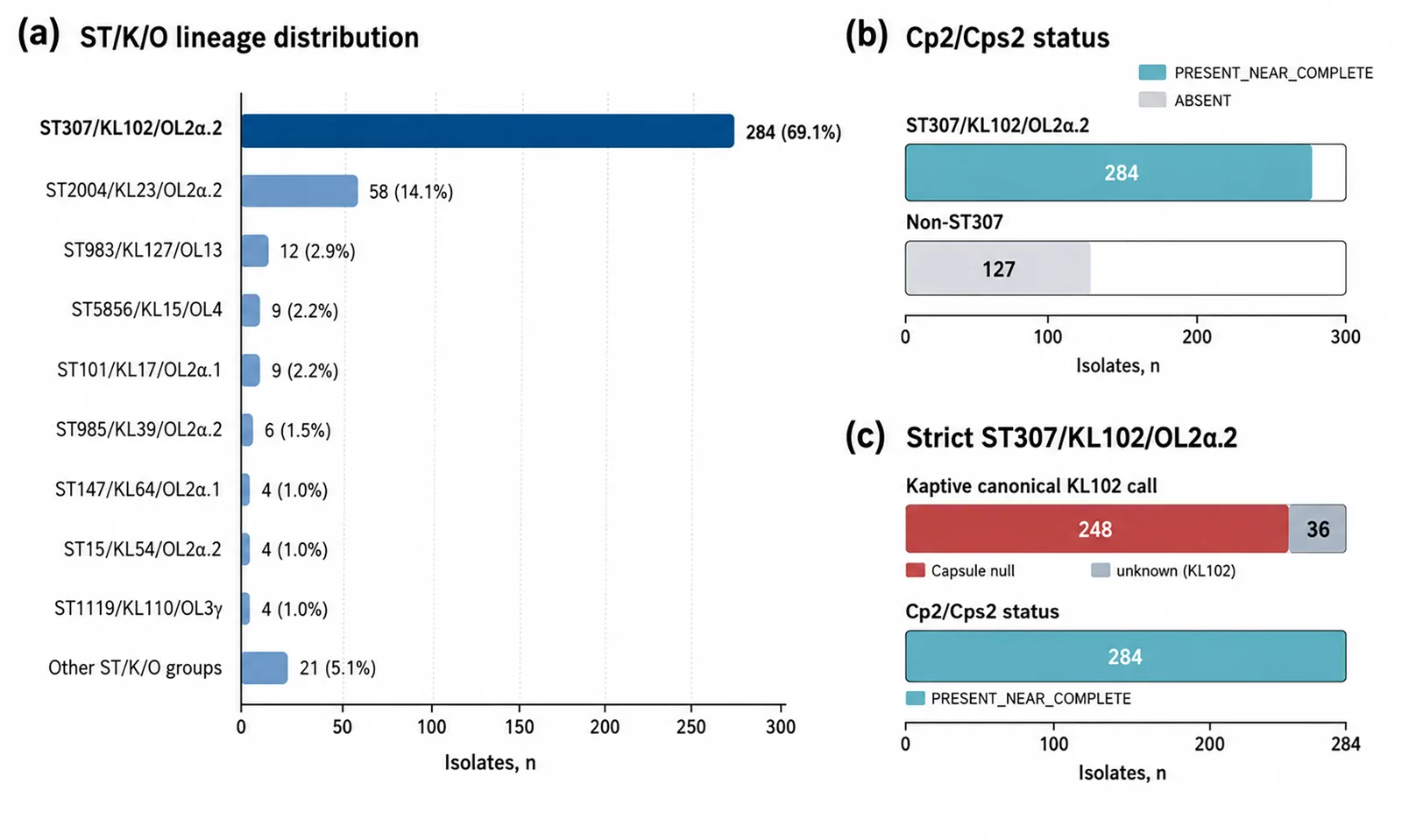
Lineage structure and Cp2/Cps2 distribution in the reanalysed 411-genome *K. pneumoniae* dataset. (**a**) Distribution of major ST, K-locus and O-locus lineage groups among 411 assemblies that passed quality control. (**b**) Cp2/Cps2 status in strict ST307/KL102/OL2α.2 isolates compared to non-ST307 isolates. Kaptive canonical KL102 calls and Cp2/Cps2 status are reported for strict ST307/KL102/OL2α.2 isolates. Values represent isolate counts, with each assembly counted once. Percentages are calculated based on the full 411-assembly dataset. Cp2/Cps2 positivity indicates near-complete detection of the Cp2/Cps2 locus by blastn screening and does not confirm phenotypic capsule expression. (**c**) Strict ST307 refers to ST307/KL102/OL2α.2 and excludes the ST307-1LV isolate. KL, K-locus; OL, O-locus; QC, quality control.

### Capsule architecture and CP2/CPS2

Within the dominant ST307/KL102/OL2α.2 lineage, canonical Kaptive typing classified the KL102 capsule locus as capsule-null in 248 of 284 genomes (87.3%) and unknown in the remaining 36 (12.7%). However, screening for the alternative polysaccharide locus Cp2/Cps2 detected it in all 284 ST307 genomes and in none of the 127 non-ST307 genomes (Fig. 2). Canonical KL102 typing alone would therefore classify most of the dominant clones as capsule-null. In contrast, the uniform Cp2/Cps2 signal indicates conserved carriage of an alternative capsule-associated locus, interpreted as coding potential rather than proof of expression. Targeted screening of the disrupted KL102 locus detected no IS overlapping the breakpoint or located within 5 kb of it in any genome, with ISNCY-family elements present only elsewhere on the same contig. Hence, the disruption was not attributable to an IS at the breakpoint (Table 1). The OL2α.2 O-locus was shared across ST307, ST2004, ST985 and ST15, while minority lineages carried OL13, OL4, OL2α.1 and OL3γ.

**Table 1.** Insertion-sequence context of the disrupted KL102 capsule locus in capsule-null ST307 genomes

Breakpoint IS-context category	Definition	Nearest IS family	Breakpoint records, n (%)	Bearing on IS-mediated truncation
**IS overlapping the breakpoint**	IS overlaps the inferred breakpoint coordinates	Not applicable	0 (0)	Would directly implicate an IS; none detected.
**IS within 5 kb of the breakpoint**	Nearest IS<=5,000 bp from breakpoint, without overlap	Not applicable	0 (0)	Would support an IS-mediated lesion; none detected.
**IS on same contig, >5 kb from breakpoint**	IS predicted on the breakpoint-bearing contig but more than 5,000 bp away	ISNCY	230 (91.6)	Not physically linked to the lesion; best treated as incidental in this analysis.
**No IS on breakpoint contig**	No IS predicted on the contig carrying the breakpoint	Not applicable	21 (8.4)	No IS present on the breakpoint-bearing contig.
**All breakpoint records evaluated**	**Not applicable**	**Not applicable**	**251** (**100**)	**No IS was detected at or near any inferred breakpoint**.

Percentages are calculated using 251 breakpoint records from capsule-null ST307 genomes. ‘Not applicable’ indicates that no nearest IS family was assigned because no IS was detected in the relevant category.

### Resistance genotypes

Most genomes carried an ESBL gene without a carbapenemase, corresponding to Kleborate resistance score 1 (396 out of 411; 96.4%), while 11 genomes (2.7%) carried a carbapenemase without a colistin-resistance gene (score 2), and 4 genomes (1.0%) lacked both ESBL and carbapenemase genes (score 0). No genome carried the combined ESBL, carbapenemase and colistin-resistance profile corresponding to score 3 (Fig. 3). The *β*-lactamase repertoire was dominated by *bla*CTX-M-15, which was detected in 406 out of 411 genomes (98.8%). ESBL positivity was nearly universal in ST307 (283 out of 284; 99.6%) and ST2004 (57 out of 58; 98.3%) and was present in all genomes of several minority lineages, including ST983, ST5856, ST340, ST147, ST985, ST15 and ST1486 (Fig. 3). Thus, ESBL carriage was widespread across the population rather than being restricted to the dominant clone. Other *β*-lactamases were also detected, including *bla*OXA-1 in 370 genomes (90.0%), *bla*SHV-28 in 286 genomes (69.6%), *blaTEM-105* (13 genomes), *bla*TEM-10 (12 genomes) and a single-genome occurrence of *bla*CTX-M-14, indicating a broader *β*-lactamase repertoire beyond *bla*CTX-M-15. However, most ESBL genotypes were attributable to *bla*CTX-M-15. Carbapenemase genes, all of which were *bla*NDM-5 (11 out of 411; 2.7%), were not detected in ST307. Instead, they were restricted to the KL15/OL4 lineages ST5856 (nine genomes) and ST340 (two genomes).

**Fig. 3.**
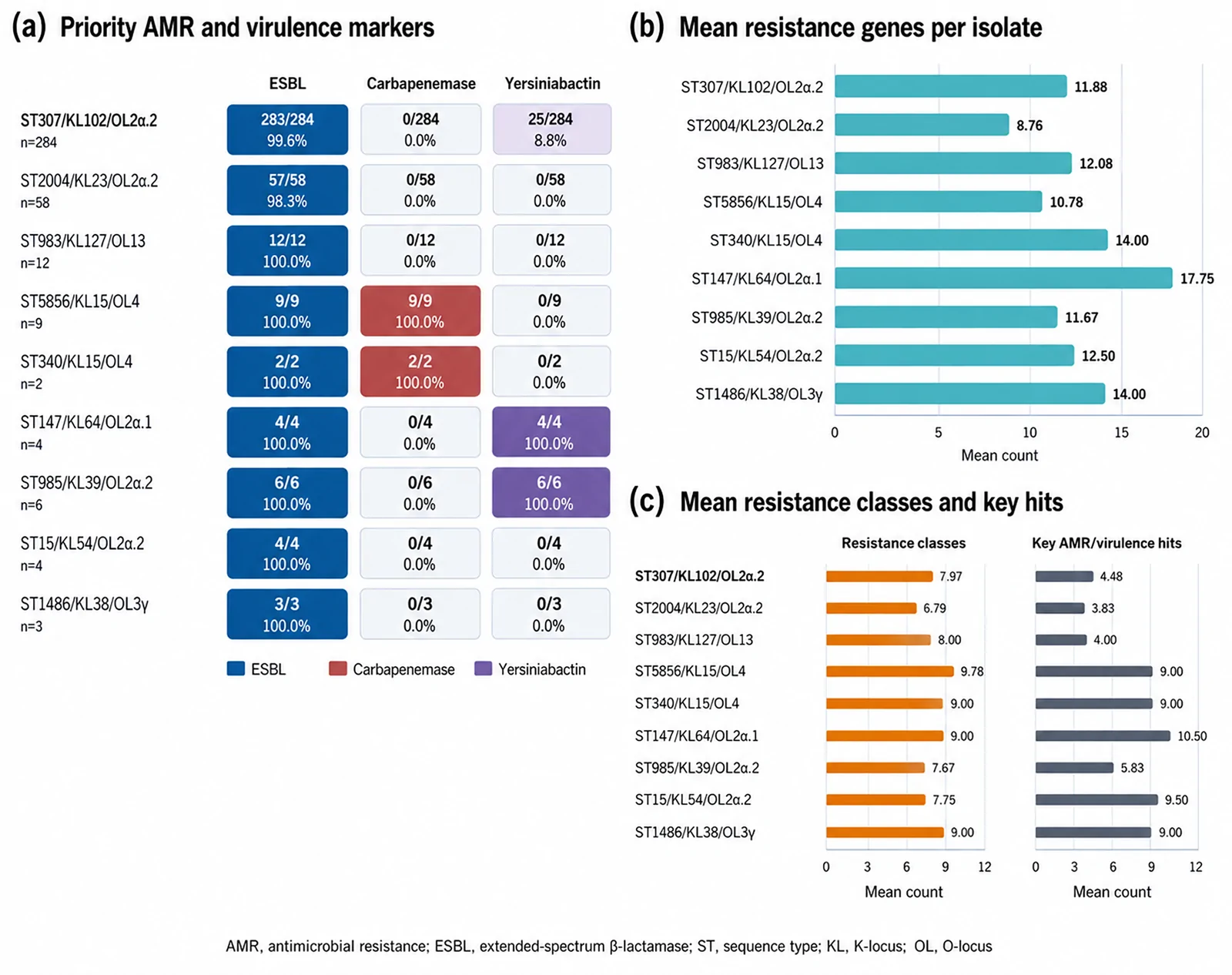
AMR and virulence-associated features among priority *K. pneumoniae* lineage groups. (**a**) Distribution of ESBL, carbapenemase and yersiniabactin signals among selected priority ST, K-locus and O-locus lineage groups. Values indicate the number of positive isolates over the total number of isolates in each lineage, with percentages provided. (**b**) Mean number of resistance genes per isolate by lineage group. (**c**) Mean number of resistance classes and key AMR or virulence gene hits per isolate by lineage group. Values are summarized from the priority lineage interpretation table, with each isolate counted once within its ST/K-locus/O-locus lineage group. KL, K-locus; OL, O-locus.

The non-*β*-lactam resistance genes identified in this population spanned multiple antibiotic classes and other antimicrobial agents (Fig. 3). Sulphonamide resistance was mainly represented by *sul2* (406 out of 411; 98.8%), with *sul1* detected in a smaller subset (51 out of 411; 12.4%). Aminoglycoside-resistance genes included *aph(3″)-Ib* and *aph(6)-Id* (formerly *strA* and *strB*) in 376 genomes each (91.5%), *aac(3)-IIa* in 312 genomes (75.9%) and *aadA2* in 42 genomes (10.2%). Trimethoprim resistance was primarily associated with *dfrA14* (350 out of 411; 85.2%) and, less frequently, *dfrA12* (40 out of 411; 9.7%). Tetracycline resistance was mainly associated with *tet(A*) (302 out of 411; 73.5%). Quinolone-associated determinants included *aac(6′)-Ib-cr* (369 out of 411; 89.8%), *qnrB1* (283 out of 411; 68.9%) and *qnrS1* (49 out of 411; 11.9%). Chloramphenicol resistance was represented by *catB3* (370 out of 411; 90.0%), while macrolide resistance was associated with the *mph(A)–mrx(A*) module (44 out of 411; 10.7%). The biocide-associated gene *qacEΔ1* was also detected in 51 genomes (12.4%).

### Chromosomal resistance determinants

Beyond the acquired resistance genes described above, the population also carried chromosomal and mutational determinants affecting fluoroquinolone and *β*-lactam susceptibilities. Fluoroquinolone resistance was also driven chromosomally by QRDR mutations in *gyrA* and *parC*. Kleborate predicted 341 of 411 genomes (83.0%) as non-wild-type resistant, 6 (1.5%) as non-wild-type intermediate and 64 (15.6%) as wild-type, the most frequent genotype being the combined GyrA p.Ser83Ile and ParC p.Ser80Ile double substitution, present in 291 of 411 genomes (70.8%) (Table S4, available in the online Supplementary Material). Outer membrane porin changes were uncommon, occurring in 21 of 411 genomes (5.1%), with the OmpK35 p.Asn116fs frameshift predominating (15; 3.6%, mainly ST307), OmpK35 p.Met2fs in ST147 (4; 1.0%), OmpK35 p.Ala243fs in 1 genome of ST307 (0.2%) and an OmpK36 deletion in ST340 (1; 0.2%), confined to these 3 lineages (Table S4).

### Resistance burden by lineage

The number of resistance genes per isolate did not simply increase with lineage size (Fig. 3). For example, ST147/KL64/OL2α.1 had the highest average number of resistance genes per isolate (17.75), while ST340 and ST1486 also had high counts (14.00 each). In contrast, the dominant ST307 lineage had a lower mean (11.88), with ST15 (12.50), ST983 (12.08) and ST985 (11.67) showing intermediate values, and ST2004 the lowest (8.76). A similar trend was seen for the number of distinct resistance classes: ST5856/KL15/OL4 carried the most (9.78), while ST307 had 7.97. The highest average burdens of key AMR or virulence determinants were observed in ST147, ST15, ST5856, ST340 and ST1486. Thus, several minority lineages matched or surpassed the dominant clone in terms of resistance per isolate.

### Plasmid localization, cluster carriage and predicted mobility

Most AMR genes in this study were located on plasmids rather than the chromosome (Fig. 4). For instance, in the dominant ST307 lineage, nearly 80% of AMR genes were plasmid-associated, and 20% were chromosomal. ST5856 showed the highest plasmid proportion (86.8%), while most other lineages had between 66 and 73% of AMR genes on plasmids. An exception was ST147, where AMR genes were evenly split between plasmids and the chromosome.

**Fig. 4.**
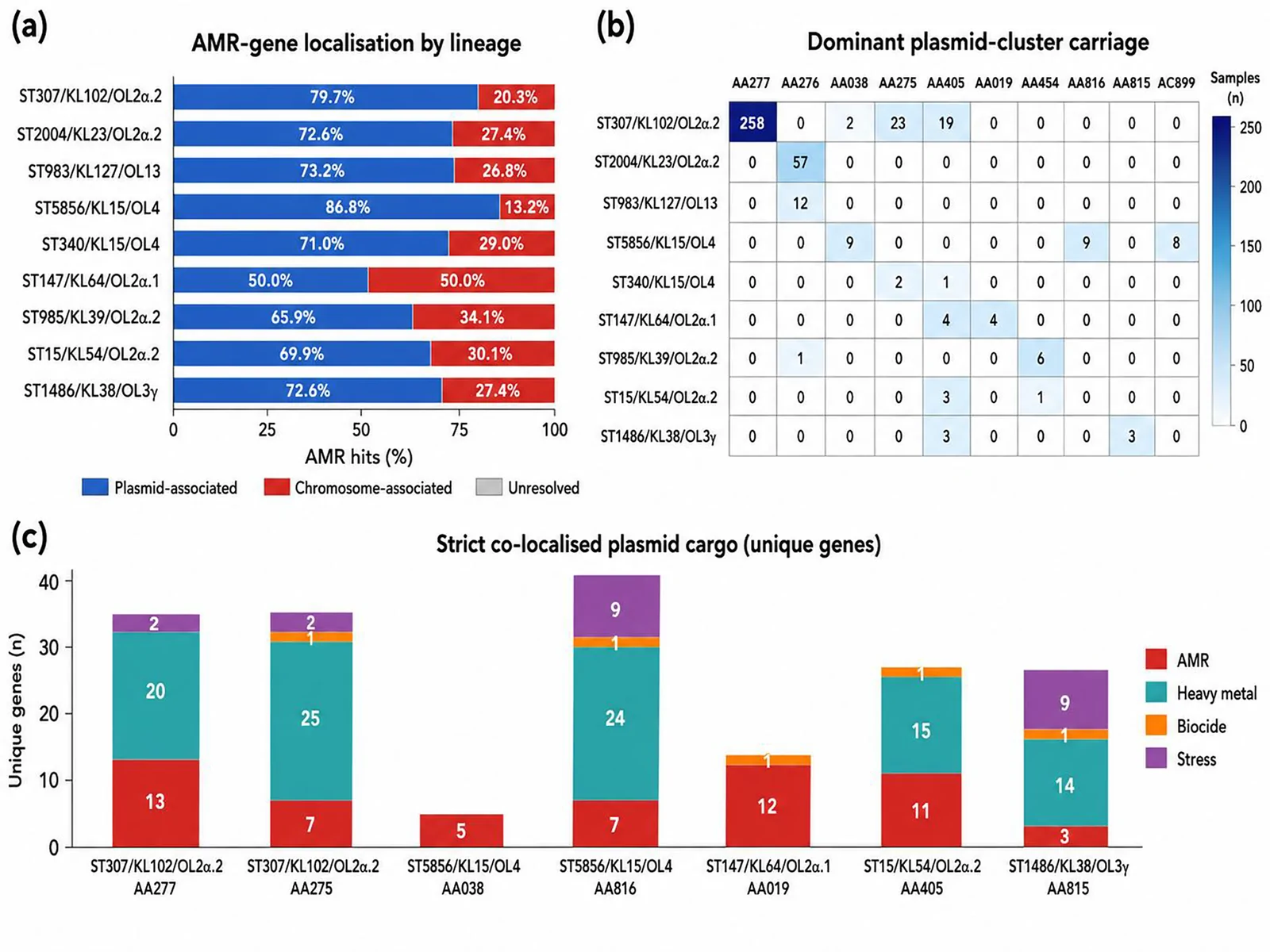
Plasmid-associated AMR architecture and co-localized plasmid cargo across priority *K. pneumoniae* lineages. (**a**) Proportional localization of AMR gene hits by lineage, classified as putative plasmid-associated, chromosome-associated or unresolved using AMRFinderPlus and MOB-suite contig-level integration. (**b**) Dominant MOB-suite plasmid-cluster carriage by lineage, with cell values indicating the number of isolates carrying each primary plasmid cluster. AA038 is included due to its carriage of carbapenemase-associated AMR signals in ST5856/KL15/OL4. (**c**) Strict plasmid-contig co-localization of cargo genes in selected lineage–cluster combinations. Genes are considered co-localized only when detected on the same MOB-suite plasmid-associated contig within the same sample. Stacked bars represent the number of distinct gene symbols in each co-localized cargo category; repeated detections of the same gene across isolates are counted once within each lineage–cluster group.

**Fig. 5.**
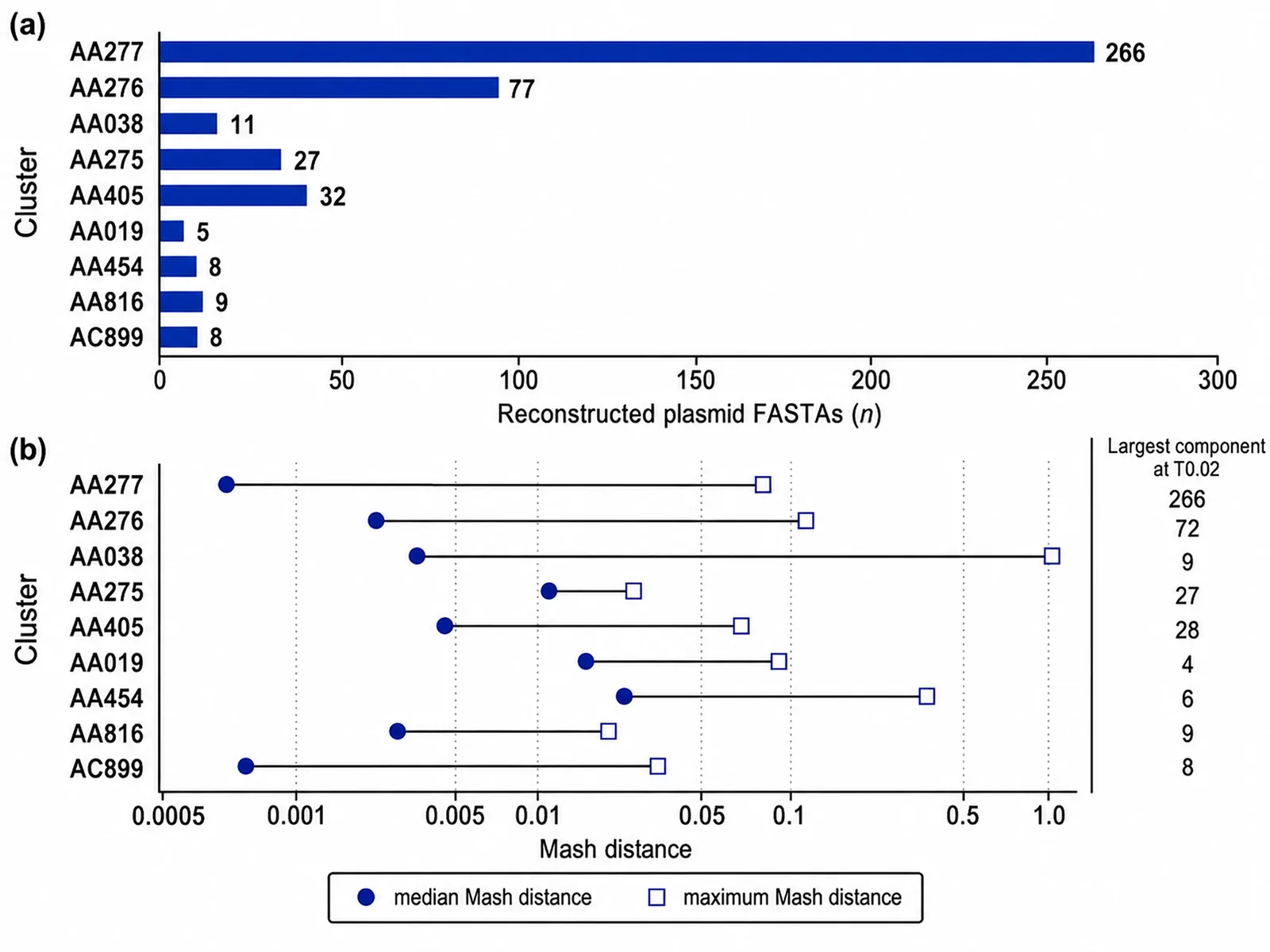
Sequence relatedness of priority plasmid clusters inferred from Mash distance analysis. (**a**) Number of reconstructed plasmid FASTA sequences analysed within each priority plasmid cluster. (**b**) Within-cluster sequence-relatedness is summarized by the Mash distance. For each cluster, the filled circle indicates the median Mash distance, and the open square indicates the maximum Mash distance observed among pairwise comparisons; the horizontal line spans the interval between these values. The right-hand column shows the size of the largest connected component at the Mash distance threshold of 0.02, indicating the largest subgroup of closely related plasmid sequences recovered within each cluster. Lower Mash distances indicate greater sequence similarity. Cluster AA277 showed the largest and most cohesive sequence set, whereas clusters such as AA038 and AA454 showed greater internal heterogeneity.

The distribution of plasmid clusters (Figs. 4 and 5) was closely linked to bacterial lineage. In ST307, cluster AA277 was the principal plasmid group (present in over 90% of ST307 genomes), with AA275 and AA405 as minor clusters. Cluster AA276 was dominant in ST2004 and across all ST983 genomes. The carbapenemase-associated cluster AA038 was present in all ST5856 genomes and two ST307 genomes; ST5856 also commonly carried clusters AA816 and AC899. Other clusters (such as AA019, AA405 and AA454) were characteristic of specific minority lineages, so each major background carried a distinctive dominant plasmid cluster.

Replicon backbones, the main plasmid structural types, were also unevenly distributed across lineages (Fig. 4). The most frequent backbone types were IncFIB (98.8%), IncFII (79.6%) and ColRNAI (67.2%). In comparison, IncY (14.4%), IncR (8.3%), IncFIA (4.1%), IncHI1B (3.4%) and IncX3 (2.2%) were less common. These backbones were lineage-structured: ST307 almost always had IncFIB and IncFII; ST2004 and ST983 were primarily IncFIB; carbapenemase-positive ST5856 carried IncFIB, IncFII, IncR and IncX3 in all genomes and IncHI1B in most. Other minority lineages had their own characteristic combinations.

Of 1,275 reconstructed plasmids, 34.3% were predicted to be conjugative (capable of self-transfer), 23.6% mobilizable (requiring help to transfer) and 42.1% non-mobilizable. Among the main plasmid clusters, those associated with ESBL were predominantly conjugative (AA277 IncFIB/IncFII, 98.1%; AA276 IncFIB, 100%; AA275, 100%; AA405, 90.6%), as was the IncX3 carbapenemase cluster AA038 (81.8%). In contrast, accessory-gene-rich clusters such as AA816 (IncHI1B) and AC899 were non-mobilizable. Some other clusters (e.g. AA019 and AA815) showed lower self-transfer potential. Importantly, these predictions are based on MOB-suite computational analysis and represent potential, not confirmed, plasmid transfer.

### Co-localized plasmid cargo

In several plasmid backgrounds, AMR genes were located on the same DNA segments (contigs) as genes conferring resistance to metals, biocides or environmental stress (Fig. 4; Table S6). For example, in ST307, the AA277 plasmid cluster typically carried 13 AMR genes, 20 metal-resistance genes and 2 stress-response genes. The AA275 cluster in the same lineage carried 7 AMR genes, 25 metal-resistance genes, 1 biocide-resistance gene and 2 stress-response genes. Similarly, in ST5856, the IncHI1B cluster AA816 contained 7 AMR genes, 24 metal-resistance genes, 1 biocide gene and 9 stress genes. A comparable pattern of metal-rich plasmid cargo was seen in ST15 (AA405) and ST1486 (AA815). In contrast, the IncX3 AA038 plasmid cluster was more streamlined, carrying five AMR genes (including *blaNDM-5*) but lacking any co-localized metal-, biocide-, stress- or virulence-related genes. Across all plasmid backgrounds, there was no instance where AMR and virulence genes were located together on the same contig.

### Nested integron and insertion-sequence context

Priority genes resolved into distinct plasmid-associated and local mobile genetic element contexts (Fig. 6; Table S7). The major resistance determinants were predominantly assigned to putative plasmid-associated contigs, including *blaCTX-M-15* in 403 out of 406 positive genomes (99.3%), *sul2* in 391 out of 406 (96.3%), the *mph(A)/mrx(A*) module in 38 out of 44 (86.4%) and *blaNDM-5* in 9 out of 11 (81.8%). Local insertion-sequence context differed by determinant. *blaCTX-M-15* was frequently IS-associated, with IS presence detected for 373 out of 406 loci (91.9%), while *blaNDM-5* showed IS presence in all 11 positive loci (100.0%). The *mph(A)/mrx(A*) module showed IS presence in 32 out of 44 loci (72.7%). Among the priority determinants, *sul2* showed the most frequent IS-flanked pattern, detected in 82 out of 406 loci (20.2%). Candidate composite-transposon-like calls were rare, occurring in only two *blaCTX-M-15* loci and two *sul2* loci, corresponding to 0.5% of positive loci for each determinant.

**Fig. 6.**
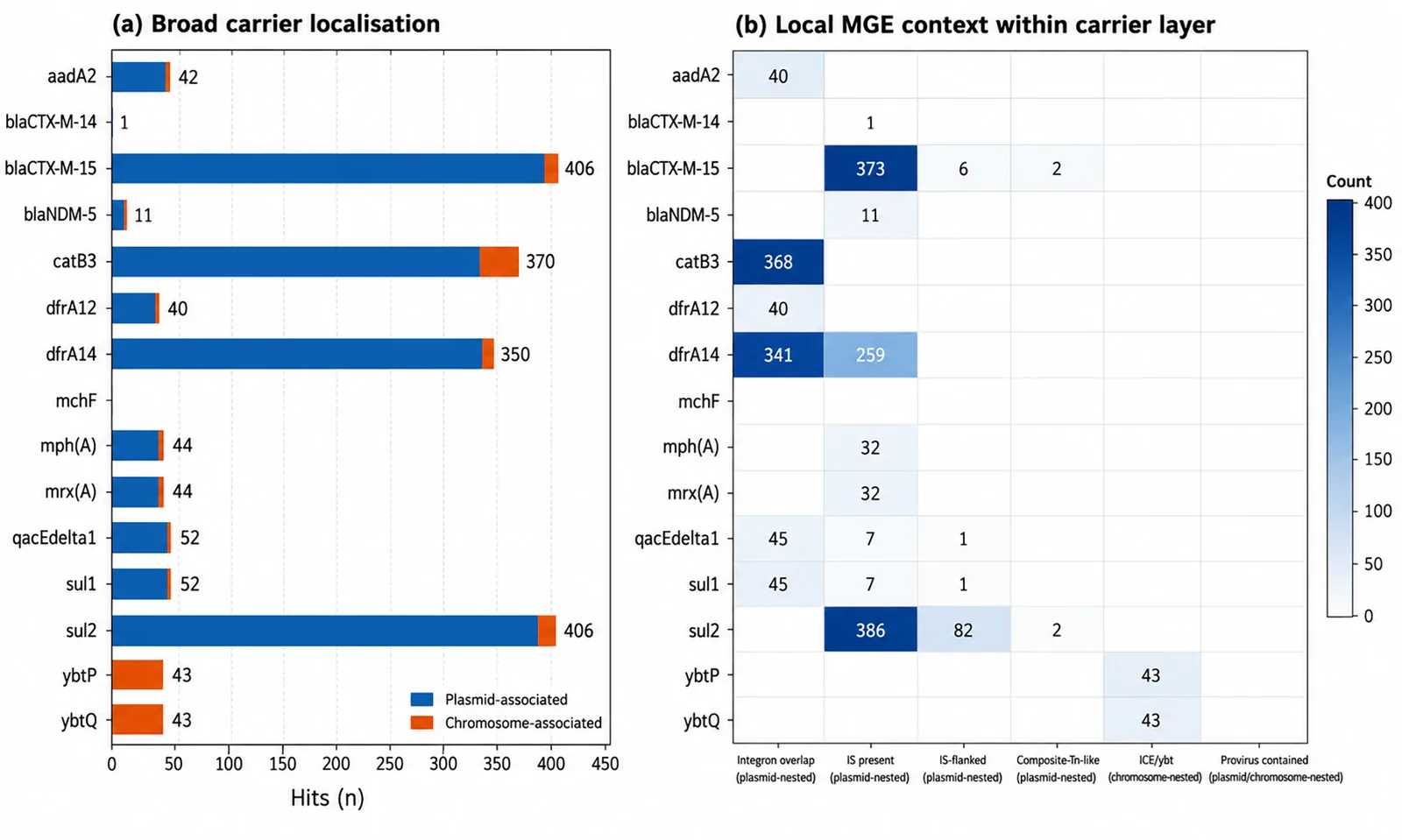
Plasmid-associated versus chromosomal localization and nested MGE context of priority genes. (**a**) Broad localization of selected AMR, biocide-associated and virulence-associated genes, indicating the number of hits assigned to putative plasmid-associated or chromosome-associated contigs based on AMRFinderPlus and MOB-suite integration. (**b**) Local MGE context of the same gene hits, displayed according to whether the context was nested within a plasmid-associated or chromosomal background. Heatmap values indicate the number of hits with integron overlap, IS presence, IS-flanked context, candidate composite-transposon-like context, ICE/ybt association or predicted provirus containment. Integron, IS, IS-flanked and composite-transposon-like signals were observed in plasmid-nested contexts, while *ybtP* and *ybtQ* were detected in chromosome-nested ICE/ybt contexts. Zero values are omitted from heatmap labels for clarity. ICE/ybt, yersiniabactin-associated integrative conjugative element context; Tn, transposon.

A separate group of determinants was characterized by direct integron or cassette overlap. Direct IntegronFinder overlap was detected for *catB3* in 368 loci, *dfrA14* in 341 loci, *dfrA12* and *aadA2* in 40 loci each and *qacEΔ1* and *sul1* in 45 loci each. These results separated the priority resistance genes into two main local organizational patterns: plasmid-associated, IS-rich contexts involving *blaCTX-M-15*, *blaNDM-5*, *sul2* and the *mph(A)/mrx(A*) module; and integron/cassette-associated contexts involving *catB3*, *dfrA* genes, *aadA2*, *sul1* and *qacEΔ1*.

### Plasmid-cluster relatedness

Priority MOB-suite plasmid clusters differed in internal sequence relatedness (Fig. 5; Table S9). AA277, the dominant ST307-associated cluster, was large and highly cohesive, comprising 266 reconstructed plasmid sequences with a median Mash distance of 0.0006, and all 266 sequences remained connected within the largest component at a distance threshold of 0.02. AC899 and AA816 were also highly cohesive at this threshold. AA276 and AA405 showed greater internal diversity, with median Mash distances of 0.0023 and 0.0052, respectively, but most sequences remained within the largest 0.02 component: 72 out of 77 for AA276 and 28 out of 32 for AA405. AA275 was more divergent, with a median Mash distance of 0.0104, but also remained connected at the 0.02 threshold.

AA038 showed a clear substructure. Although its median Mash distance was 0.0028, the maximum distance was 1.0, indicating divergent reconstructions within the cluster. At the 0.02 threshold, AA038 comprised a coherent nine-sequence ST5856-associated *blaNDM-5* subgroup and two divergent reconstructed plasmid sequences that were separated from this subgroup. AA454 was the most heterogeneous priority cluster, with a median Mash distance of 0.0178, a maximum distance of 0.296 and only 6 out of 8 sequences in the largest 0.02 component. AA019 was also relatively diverse, with a median Mash distance of 0.0137 and 4 out of 5 sequences retained in the largest 0.02 component.

### Virulence loci and prophage context

The virulence-locus landscape was restricted across the dataset. Most genomes had a Kleborate virulence score of 0 (368 out of 411; 89.5%), while 43 out of 411 genomes (10.5%) had a score of 1; no genome had a virulence score of 2–5. No aerobactin, salmochelin, colibactin or Rmp-associated loci were detected. Yersiniabactin was detected in 43 out of 411 genomes (10.5%) and showed a lineage-associated distribution, occurring in 25 out of 284 ST307/KL102/OL2α.2 genomes (8.8%) and in all ST147/KL64/OL2α.1 (4 out of 4) and ST985/KL39/OL2α.2 (6 out of 6) genomes, but not in the other major lineage profiles (Fig. 3; Tables S2 and S3). The yersiniabactin-associated markers *ybtP* and *ybtQ* were chromosome-associated in all 43 positive genomes and assigned to an ICE/ybt-linked context, separating the principal virulence signal from the plasmid-associated AMR architecture (Fig. 6; Table S7).

Predicted prophage/provirus regions were widespread, with 1,559 provirus intervals detected across 410 out of 411 genomes (99.8%), corresponding to a mean of 3.8 intervals per genome (Table S8). However, strict coordinate-overlap analysis did not classify any priority AMR or virulence determinant as provirus-contained (Fig. 6; Tables S7 and S8). Thus, although prophage/provirus regions were common in the dataset, they did not directly overlap the priority resistance or virulence genes analysed here.

## Discussion

This secondary genomic analysis is consistent with the UTH neonatal *K. pneumoniae* outbreak having been dominated by clonal expansion of a single high-risk lineage, ST307/KL102/OL2α.2, within a broader population of clinically significant minority lineages. The primary SPINZ study resolved strain epidemiology and transmission and reported ST307 dominance, near-universal ESBL carriage and a small carbapenemase-positive minority, on which this work builds [1]. The genomes analysed here were sampled across the pre- and post-implementation periods of the infection-prevention bundle evaluated in that study, and the present analysis treats them as a single cross-section rather than attributing structure to any phase of the intervention. It extends the primary work by resolving the mobile-element architecture of the same population. To the author’s knowledge, this is the first integrated clone-, capsule-, plasmid- and mobile-element-resolved reanalysis of Zambian neonatal *K. pneumoniae*.

### ST307 as a local form of a global problem

The dominance of ST307 is best read as a local manifestation of a global phenomenon. ST307 spread internationally before formal recognition and is now an established ESBL high-risk clone associated with *blaCTX-M-15* and IncF plasmids [35, 36, 59]. The UTH clone displayed the ESBL-based resistance profile typical of ST307 but, unlike ST307 in many high-income settings, had not acquired carbapenemase genes. Such acquisitions, including KPC, OXA-48-like and NDM enzymes, are well documented in ST307 from high-income countries [50, 60, 61]. ST307 variants carrying *blaOXA-181* alongside ESBLs have been reported in southern Africa [62, 63]. Moreover, MDR-hypervirulent ST307 has been described in eastern Africa [64]. Several minority lineages also carried high-risk features, including the globally important clones ST147 and ST101 [37, 65] and the KL15/OL4 lineages ST5856 and ST340, the latter in clonal complex 258, which harboured carbapenemase genes [1, 35]. ST2004, the second most common lineage, remains poorly characterized [1]. The OL2α.2 O-locus shared across ST307, ST2004, ST985 and ST15 indicates a common LPS background which, because O-antigen structure influences complement-mediated serum killing, may be relevant to bloodstream survival, although serum-resistance phenotypes were not tested [66].

In sub-Saharan Africa, ESBL-producing *K. pneumoniae* occurs in both polyclonal forms, reported from Malawi and hospitals across East and West Africa [67–70], and single-clone outbreaks, including ST14 neonatal sepsis in Tanzania [10], a neonatal outbreak in Malawi [11] and further clonal ESBL *K. pneumoniae* in Tanzanian neonatal units [71]. The UTH outbreak sits at the clonal end of this spectrum. However, its minority lineages still contribute resistance genes, plasmids and virulence factors that add diversity and a continued regional threat [12, 72].

### Structure of the resistome

The structure of the resistome is informative. The ESBL signal in this population was overwhelmingly attributable to a single allele, *blaCTX-M-15*, with little contribution from other ESBL families. This is more consistent with clonal expansion of a dominant ST307 background than with repeated independent acquisition of different ESBL enzymes across diverse lineages, an interpretation supported by the clonal structure reported in the primary study [1]. It also mirrors the global ST307 lineage, in which *blaCTX-M-15* is the hallmark of MDR expansion and is most often carried on IncF-type plasmid backbones that frequently co-carry non-*β*-lactam determinants [35, 36, 50]. The concordant distribution of *blaOXA-1* and *aac(6′)-Ib-cr* is consistent with carriage in a linked multiresistance region, as reported in CTX-M-15-producing *K. pneumoniae* internationally [50, 73], and the non-ESBL *blaSHV-28* allele tracked with the ST307 background, consistent with a clone-associated chromosomal *β*-lactamase rather than a recently acquired ESBL.

This organization extended beyond the *β*-lactamases, with non-*β*-lactam determinants arranged into recurrent packages rather than appearing as isolated acquisitions. The dominant pattern was a plasmid-associated backbone comprising *sul2*, *aph(3″)-Ib–aph(6)-Id*, *dfrA14*, *tet(A*), *aac(3)-IIa* and *qnrB1*, alongside the near-universal *blaCTX-M-15* background. A second block corresponded to class 1 integron-associated determinants, comprising the cassette genes *dfrA12* and *aadA2* together with the conserved-region markers *sul1* and *qacEΔ1*. A lower-frequency *mph(A)/mrx(A*) macrolide module formed a further package, concordant with the primary study, in which the macrolide determinant in ST307 sublineages could not be fully resolved from short reads but was inferred to be plasmid-borne [1]. These modules were treated as features of the population as a whole; the primary study reported that some content, including the macrolide determinant and parts of the class 1 integron complement, varied across ST307 sublineages rather than being uniform across the clone [1], which the present cross-sectional framing does not resolve. Taken together, these patterns indicate that the UTH resistome was not a random collection of independent genes but a limited number of recurrent, largely co-inherited plasmid-, integron- and insertion-sequence-associated units. This is consistent with the established view that multidrug resistance in Enterobacterales, including *K. pneumoniae*, is assembled and maintained through linked mobile-element structures rather than the independent acquisition of single genes [25, 26, 30]. Clinically, such an organization implies that resistance to several drug classes may be co-selected or co-maintained when the clone or plasmid background carrying them expands. However, short-read assemblies cannot confirm physical linkage, and long-read closure, transfer experiments and phenotypic testing would be required to establish transferability and treatment-level consequences.

### A nested plasmid, integron and insertion-sequence architecture

The plasmid analysis resolved this organization across nested genetic contexts rather than as a simple inventory of resistance genes (Fig. 6; Table S7). Several determinants overlapped IntegronFinder intervals, of which *catB3*, *dfrA14*, *dfrA12* and *aadA2* are consistent with integron-associated cassette cargo, whereas *sul1* and *qacEΔ1* are better read as conserved-segment markers of class 1 integrons. This matters because integrons capture and rearrange cassettes over time, allowing plasmid-associated platforms to evolve beyond single-gene acquisition [25].

A second group of determinants lies outside direct integron overlap in insertion-sequence-associated contexts, best read as family-level signals rather than complete reconstructions of transposition events. *blaCTX-M-15* was frequently associated with IS1380- or IS91-family elements, consistent with the role of ISEcp1-like elements of the IS1380 family in *blaCTX-M* mobilization, although short reads did not resolve complete ISEcp1–*blaCTX-M-15* structures [18, 74]. *blaNDM-5* was IS-proximal in all positive genomes, most often with IS5-family elements, compatible with the contexts characteristic of NDM mobilization, though without resolving the bounding element. *sul2* showed the most frequent IS-flanked configuration, and the strongest candidate composite structures for *blaCTX-M-15* involved IS6-family elements, which include IS26. African comparator data support this interpretation, showing that *blaCTX-M-15* environments on the continent commonly involve ISEcp1, class 1 integrons and IncF plasmids, including Tanzanian *K. pneumoniae* carrying *blaCTX-M-15* on conjugative IncF plasmids [10, 75]. These findings indicate locally structured resistance regions within plasmid backgrounds while stopping short of demonstrating complete transposition units.

A central implication is that repeated detection of the same resistance gene need not indicate spread of a single unchanged plasmid. Here, *blaCTX-M-15* was near-universal yet occurred within more than one plasmid background, most notably the IncFIB/IncFII-associated cluster AA277 in ST307 and the IncFIB-associated cluster AA276 in ST2004 and ST983 (Fig. 5; Tables S5 and S9). The AA-cluster assignments derive from the present MOB-suite analysis. AA277 was highly related but internally variable across ST307 genomes, consistent with a recurrent clone-associated plasmid background carried through ST307 expansion rather than a single intact plasmid, with the caveat that short-read fragmentation can inflate apparent divergence. A comparable pattern, a conserved dominant IncFIB(K) backbone coexisting with more divergent variants, has been reported for clone-associated plasmids in East African ESBL-*K. pneumoniae* using the same distance-based approach [64]. AA276 represented a second, more internally diverse IncF background, and East African neonatal and One Health studies support the dissemination of *blaCTX-M-*15 through multiple conjugative IncF plasmids rather than a single universal structure [10, 75].

### The carbapenemase plasmid background and convergence risk

In contrast to the IncF-associated ESBL backgrounds that dominated the population, the carbapenemase-positive minority showed a distinct and clinically important pattern. *blaNDM-5* was restricted to the KL15/OL4 lineages ST5856 and ST340. In ST5856, it was plasmid-associated on the IncX3 cluster AA038, whereas in ST340, it was chromosome-associated, without AA038 or IncX3 (Fig. 5; Tables S5 and S9). The AA038 backbone also occurred, without *blaNDM-5*, in two ST307 genomes (Fig. 4), so the genetic background for convergence is already present [76]. The occurrence of the same cluster in both lineages is consistent with a shared carbapenemase plasmid background. However, short reads cannot establish the direction, timing or setting of transfer. Regional experience sharpens its relevance. In East Africa, *blaNDM-5* is already established in the Enterobacterales ecology, including neonatal *Escherichia coli* co-carrying it with *blaCTX-M-15* on IncFIA plasmids and a carbapenem-resistant *K. pneumoniae* carrying *blaNDM-5* and *blaOXA-181* on distinct IncX3 backgrounds [77, 78]. Southern African data provide a closer warning, documenting ST307 carrying IncX3-associated *blaOXA-181*, the movement of IncX3 carbapenemase plasmids into other lineages and neonatal unit outbreaks of carbapenemase-producing *K. pneumoniae* [62, 63, 79, 80]. The concern at UTH is therefore not that carbapenem-resistant ST307 was already present but that convergence between the dominant ESBL-producing ST307 population and an IncX3-*blaNDM-5* plasmid is plausible. NDM-5-producing ST307 has emerged independently elsewhere [81, 82], and such convergence would offer a credible route to carbapenem-resistant ST307, which could sharply narrow empirical options for neonatal sepsis, where rapid diagnostics and reserve agents are scarce.

### Predicted mobility and accessory cargo

Predicted mobility added a prioritization layer. The priority clusters were enriched for predicted-conjugative plasmids, including the ST307 ESBL cluster AA277, the ST2004/ST983 ESBL cluster AA276 and the IncX3 *blaNDM-5* cluster AA038 (Fig. 5; Tables S5, S9 and S10). This links abundant or high-risk lineages to plasmid backgrounds carrying major resistance modules with the potential for transfer, but the calls are genomic prioritization signals rather than evidence of transfer.

Accessory cargo analysis showed that several plasmid backgrounds carried more than resistance genes alone (Fig. 4; Table S6). On the same-contig analysis, the ST307-associated IncF background AA277 and the cargo-rich IncHI1B-associated ST5856 background carried resistance determinants together with heavy-metal, biocide or stress-response genes, whereas the IncX3-associated *blaNDM-5* cluster AA038 appeared comparatively lean. This suggests that the ESBL-associated IncF and IncHI1B backgrounds favour long-term persistence in the hospital niche, while the IncX3-*blaNDM-5* background appears specialized for carbapenemase carriage and mobility. Plasmids co-carrying antibiotic-resistance and metal- or biocide-tolerance determinants can persist under non-antibiotic selection such as disinfectant or metal exposure [83–85], relevant in a neonatal unit where disinfection and antiseptic use are central to infection prevention, and the integron-associated *qacEΔ1* signal adds to this, given its link to reduced quaternary-ammonium-compound susceptibility [25, 86]. These interpretations remain cautious, since same-contig co-localization in short-read assemblies demonstrates co-occurrence rather than functional co-selection, and southern African data caution that gene carriage does not always translate into measurable disinfectant resistance [87].

### The chromosomal compartment

The plasmid-associated resistome takes on meaning alongside chromosome-derived features, since resistance and virulence emerge from interactions among distinct genomic compartments [40]*. K. pneumoniae* carries a chromosomal class A SHV *β*-lactamase, explaining its intrinsic aminopenicillin resistance [88], and here, the non-ESBL blaSHV-28 allele is associated with the dominant ST307 population. Hence, the ampicillin component of standard neonatal therapy is intrinsically weak, while the accompanying aminoglycoside may be undermined by acquired determinants such as *aac(3)-IIa*. Mutational modifiers added to this: QRDR substitutions occurred alongside plasmid-mediated quinolone-resistance genes, underlying the high predicted burden of fluoroquinolone non-wild-type profiles. Porin changes were less frequent, but their importance may exceed their prevalence, since disruption of OmpK35 or OmpK36 can reduce *β*-lactam influx and contribute to carbapenem non-susceptibility when combined with ESBL or carbapenemase activity [89, 90]. The OmpK36 alteration in ST340 is notable because that lineage also carried *blaNDM-5*, a genotype in which carbapenemase production and reduced permeability could reinforce one another. However, this is tentative, as carbapenem MICs and porin expression data were unavailable.

The same compartmental logic applies to virulence, which here was distinct from classical hvKp. Nearly all genomes lacked major hypervirulence loci; the only recurrent marker was ICEKp-linked yersiniabactin, while aerobactin, salmochelin, colibactin and *rmp* were absent (Fig. 3; Table S7) [4, 40]. This argues against widespread convergence of resistance and virulence and contrasts with an emerging trend in which ST307 acquires convergent or hypervirulent features, including an iron-acquisition-enhanced German clone, *mcr-8.1*-carrying MDR-hypervirulent ST307 and MDR-hypervirulent ST307 in East African surveillance [61, 64, 91]. The UTH population, therefore, represents a point of divergence from that trajectory rather than evidence that ST307 is intrinsically avirulent, since the background for convergence remains present. Yersiniabactin was present at low frequency in ST307 but fixed in the smaller ST147 and ST985 lineages and should not be read as a hypervirulence marker given its wide distribution and weak association with the phenotype [40]. These lineages instead represent co-occurrence of AMR and yersiniabactin, of particular concern in the globally high-risk ST147, where ESBL carriage, the highest per-isolate resistance burden in the dataset, and fixed yersiniabactin together justify clone-level concern. Crucially, yersiniabactin markers were chromosomal and ICEKp-linked in every positive genome. In contrast, the major resistance determinants were plasmid-associated and embedded in insertion-sequence or integron contexts, placing resistance and virulence in separate genomic compartments. Malawian data provide a comparator, with ESBL-producing *K. pneumoniae* distributed across diverse lineages, whereas the UTH outbreak concentrated resistance within a far more strongly ST307-dominated population [67].

### Capsule architecture, prophages and a tiered model of resistance assembly

The capsule findings refine the surface biology of the dominant lineage. As the principal virulence factor of *K. pneumoniae*, the capsule mediates serum resistance and anti-phagocytic protection, thereby underpinning survival in the bloodstream [3, 4]. The primary study reported disruption of the canonical KL102 locus by a 661 bp deletion that truncates *wbaP* and *wzy*, with retention of a second capsule-associated locus, Cp2 [1]. Consistent with this, conserved near-complete Cp2/Cps2 carriage was found across the strict ST307 background despite capsule-null or uncertain KL102 calls, and the breakpoint screen detected no IS within 5 kb of the lesion (Table 1). The capsule-null call, therefore, reflects this defined deletion rather than the insertion-sequence-mediated truncation that switches capsule states elsewhere [92], making altered architecture more plausible than a complete loss, although Cp2/Cps2 detection reflects coding potential rather than expression. Capsule type also carries epidemiological weight, since only a small subset, particularly K1 and K2 and, to a lesser extent, K5, K20 and K54, is closely linked to hypervirulence [3, 93]. None of the major UTH lineages carried these; the only exception was KL54 in the four-genome ST15 lineage, which nonetheless lacked the aerobactin, salmochelin and *rmp* loci required for hypervirulence [40]. The invasive neonatal disease seen here is therefore better explained by the inherent serum resistance of encapsulated *K. pneumoniae* in a vulnerable host with low complement activity and limited opsonic antibody, allowing a transmissible MDR clone such as ST307 to invade without classical hypervirulence determinants. At the same time, the capsule supports biofilm formation and environmental persistence, which sustain transmission independently of hypervirulence.

The prophage analysis is an informative negative result. Predicted prophages were near-universal, consistent with the high and diverse prophage content described for the species [29, 58]. However, none of the priority resistance or virulence genes lies within a predicted provirus. Because prophages are not inert and can carry resistance and shape fitness in context-dependent ways [94], this does not render them unimportant, but it shows here that they were not the vehicle for the priority determinants, consistent with a *K. pneumoniae* resistance mobilome that is plasmid-, integron- and insertion-sequence-driven.

Taken together, these observations support a tiered model of resistance assembly beyond the clonal-versus-horizontal dichotomy. Clonal expansion amplified a Cp2/Cps2-positive ST307 lineage carrying a recurrent IncF AA277 ESBL plasmid, while minority lineages accumulated distinct architectures, including the IncX3 AA038 *blaNDM-5* platform of the KL15/OL4 lineages and the AMR-yersiniabactin co-occurrence of ST147 and ST985. Within the plasmid layer, resistance genes were organized into integron cassettes and IS-flanked modules. They were often shared among plasmids with metal-, biocide- and stress-response cargo. At the same time, the principal virulence signal was chromosomal and ICE-linked, and prophages did not carry the priority genes. Clonal and horizontal processes therefore acted together, on different genomic compartments, to shape this outbreak’s resistance.

### Clinical and surveillance implications

These genotype-based findings have implications for empirical neonatal therapy, although their predictions pertain to resistance phenotypes rather than to confirmed resistance, as no susceptibility testing was performed [95]. The *blaCTX-M-15* genotype predicts resistance to penicillins and third-generation cephalosporins; *aac(3)-IIa* is expected to compromise the first-line neonatal aminoglycoside; *aac(6′)-Ib-cr* and *qnr* genes together with QRDR substitutions predict reduced fluoroquinolone susceptibility; and the frequent *blaOXA-1*, which commonly confers piperacillin-tazobactam resistance, would undermine that carbapenem-sparing option. The WHO first-line regimen of ampicillin and gentamicin with cephalosporin escalation is therefore likely to be compromised, consistent with reports from low-resource neonatal units where Gram-negative resistance outpaces accessible treatment and contributes to mortality [2, 7, 13, 96]. This leaves carbapenems as the practical last line, which is why the minority *blaNDM-5* signal is disproportionately important, since it threatens the one class for which no resistance determinant was detected in the remaining lineages.

For surveillance, these findings indicate that detecting an ESBL or carbapenemase gene is necessary but not sufficient, because its significance depends on genomic context. Clone identity, plasmid background, capsule architecture and mobile-element context would ideally be tracked alongside gene detection to anticipate where resistance may concentrate and escalate [97, 98]. Carbapenem-resistance determinants were confined to the KL15/OL4 lineages and absent elsewhere, identifying carbapenems as the agents most in need of protection and arguing for strengthened infection prevention and antimicrobial stewardship alongside continued genomic monitoring. Such monitoring should prioritize the recurrent clone and plasmid combinations identified here, namely ST307 with AA277, ST2004 and ST983 with AA276 and KL15/OL4 with AA038, and watch in particular for movement of the IncX3 *blaNDM-5* plasmid into the resident ST307 clone, the single change most likely to exhaust empirical treatment options in this vulnerable population. Realizing this will require investment in local genomic and long-read sequencing capacity, so that the structures inferred here can be closed and confirmed.

## Limitations

Several limitations qualify these interpretations. Most fundamentally, this is a secondary analysis of short-read assemblies, which cannot fully resolve complex plasmids, repetitive ISs, transposon boundaries or capsule-locus structure. The MOB-suite clusters, replicon assignments, Mash distances, predicted mobility and same-contig co-localizations are therefore computational inferences about context and mobility potential rather than proof of complete structure, conjugation, transmission or functional co-selection. The study is single-centre, does not link genomes to individual neonatal outcomes, lacks a contemporaneous non-outbreak baseline and inherits its sampling from the primary study. Because the isolates were sampled over the intervention period of the primary study and analysed as a single cross-section, the structural features described here cannot be disentangled from any temporal or selection-driven change over that period, and no feature is attributed to a phase of the infection-prevention bundle. Because no unit-level data on antimicrobial, metal or biocide usage were available, the co-selection potential identified here could not be tested against actual selective pressures. All inferences remain genotype-based, so that confirmation would require phenotypic susceptibility, conjugation, serum resistance and capsule expression assays, together with long-read sequencing.

## Supplementary material

10.1099/acmi.0.001164.v4 Supplementary Material 1.

## Abbreviations

AMR: antimicrobial resistance
ESBL: extended-spectrum *β*-lactamase
hvKp: hypervirulent *Klebsiella pneumoniae*
Inc: incompatibility
ISs: insertion sequences
KL102: K-locus 102
LMICs: low- and middle-income countries
MDR: multidrug-resistant
MGE: mobile genetic element
NDM: New Delhi metallo-*β*-lactamase
QRDR: quinolone resistance-determining region
SPINZ: Sepsis Prevention in Neonates in Zambia
STs: sequence types
WGS: whole-genome sequencing
WHO: World Health Organization
